# Transcriptome Analysis Reveals a Gene Expression Pattern Associated with Fuzz Fiber Initiation Induced by High Temperature in *Gossypium barbadense*

**DOI:** 10.3390/genes11091066

**Published:** 2020-09-10

**Authors:** Gongmin Cheng, Longyan Zhang, Hengling Wei, Hantao Wang, Jianhua Lu, Shuxun Yu

**Affiliations:** 1 College of Agronomy, Northwest Agriculture and Forestry University, Yangling 712100, China; cgm@nwafu.edu.cn; 2 State Key Laboratory of Cotton Biology, Institute of Cotton Research, Chinese Academy of Agricultural Sciences, Anyang 455000, China; nxyb@hebau.edu.cn (L.Z.); henglingwei@163.com (H.W.); wanghantao@caas.cn (H.W.); lujianhua@caas.cn (J.L.); 3 College of Agronomy, Hebei Agricultural University, Baoding 071001, China

**Keywords:** *Gossypium barbadense*, high temperature, fuzz initiation, RNA-seq, gene expression

## Abstract

*Gossypium barbadense* is an important source of natural textile fibers, as is *Gossypium hirsutum*. Cotton fiber development is often affected by various environmental factors, such as abnormal temperature. However, little is known about the underlying mechanisms of temperature regulating the fuzz fiber initiation. In this study, we reveal that high temperatures (HT) accelerate fiber development, improve fiber quality, and induced fuzz initiation of a thermo-sensitive *G. barbadense* variety L7009. It was proved that fuzz initiation was inhibited by low temperature (LT), and 4 dpa was the stage most susceptible to temperature stress during the fuzz initiation period. A total of 43,826 differentially expressed genes (DEGs) were identified through comparative transcriptome analysis. Of these, 9667 were involved in fiber development and temperature response with 901 transcription factor genes and 189 genes related to plant hormone signal transduction. Further analysis of gene expression patterns revealed that 240 genes were potentially involved in fuzz initiation induced by high temperature. Functional annotation revealed that the candidate genes related to fuzz initiation were significantly involved in the asparagine biosynthetic process, cell wall biosynthesis, and stress response. The expression trends of sixteen genes randomly selected from the RNA-seq data were almost consistent with the results of qRT-PCR. Our study revealed several potential candidate genes and pathways related to fuzz initiation induced by high temperature. This provides a new view of temperature-induced tissue and organ development in *Gossypium barbadense*.

## 1. Introduction

Mature cotton seeds are generally covered with two types of fibers (lint and fuzz), both of which are mainly composed of cellulose. The development of cotton fibers can be divided into four overlapping stages: Fiber initiation, elongation, thickening of secondary cell walls, and dehydration, which are defined on the basis of the number of days post-anthesis [1]. Lint fiber initiation occurs from −3 dpa and continues to 3 dpa [2]. With the scanning electron microscope (SEM) and cotton fiber mutants, the fuzz fiber initiation stage was confirmed at 4 dpa [3,4]. It has been reported that the first three stages of fiber development are hypersensitive to environmental stress [5]. Extreme temperatures and other abiotic stresses during fiber development can significantly reduce cotton fiber yield and quality, and may even lead to falling bolls and squares [6,7,8].

In plants, studies of trichomes and root hairs in *Arabidopsis thaliana* have dramatically expanded our understanding of cotton fiber initiation and elongation. Numerous genes have been found to be involved in trichome initiation in the model plant. In *Arabidopsis*, it has been widely believed that the MYB–bHLH–WD40 (MBW) transcriptional complex (GL3/EGL3-GL1-TTG1) determines the fate of trichome cells by inducing the expression of *GL2* [9]. In addition, *GIS2*, *ZFP8*, *ZFP6*, and *ZFP5* were found to promote the expression of MBW complex members in *Arabidopsis* [10,11,12]. There are many similarities between cotton fibers and *Arabidopsis* trichomes in the regulatory mechanism of initiation development, which has been proved by some homologous genes in cotton. *GhMYB25-like* (*GhMML3*) and *GhMYB25* (*GhMML7*) are two R2R3-MYB genes playing a significant role in regulating epidermal cell-specific differentiation and fiber initiation [13,14,15]. The silencing of *GhMYB25-like* will inhibit cotton lint and fuzz fiber initiation [13]. Overexpression of *GhMYB25* in cotton increases the number of trichomes on leaves and reduces the number of fiber cells on the ovule epidermis [15]. As another R2R3 MYB transcription factor gene, *GhMYB109* is also explicitly expressed during lint initiation and elongation development [16]. Overexpression of an HD-ZIP transcription factor gene, *GhHD1*, increased the number of initiating fibers, but had no other adverse effects on leaf hairs [17]. Cotton *PROTODERMAL FACTOR1* gene (*GbPDF1*) is mainly expressed during fiber initiation and early elongation stages, and the silencing of *GbPDF1* will retard fiber initiation [18]. Recently, a gene named *GaGIR1* has been cloned from fuzzless *G. arboreum* accessions, which may play a role in suppressing the fuzz fiber initiation [19,20]. However, genes that interact with temperature to regulate the lint or fuzz fiber initiation have not been reported.

Cotton fiber initiation and elongation are not only dependent on their genetic specificity, but also regulated by environmental factors and endogenous hormones. Auxin, gibberellin, and brassinolide have long been known to play important roles in plant cell expansion and elongation [21]. As another phytohormone, ethylene has the ability to regulate root hair and hypocotyl development in *Arabidopsis* [22,23]. Auxin, gibberellin, brassinolide, and ethylene have positive effects on cotton fiber development, while abscisic acid and cytokinin inhibit the fiber cell development [24,25,26]. Jasmonic acid (JA) increases the number of leaf trichomes in *Arabidopsis* by promoting the expression of *GL3* [27,28]. In cotton, JA-related metabolism promotes fiber initiation [29]. In addition to phytohormones, intracellular calcium (Ca^2+^) and hydrogen peroxide (H_2_O_2_) also regulate cotton fibers development [21].

Temperature is a major environmental factor affecting plant growth and development. High-temperature stress is common to all crops that originated in the tropics and subtropics. During the flowering period, high-temperature stress can cause male sterility in some crops, such as rice and cotton [30,31]. Some molecular mechanisms of plant response to high temperatures have been revealed, including the response of transcription factors and heat shock proteins, calcium ion and reactive oxygen species (ROS) signal transduction, and the response of endogenous plant hormones, such as auxin and gibberellin [32,33,34]. These studies provide a comprehensive model for the temperature-regulated development of specific tissues or organs, such as the development of cotton fuzz fibers in high temperatures.

Most *Gossypium barbadense* varieties have a low density of fuzz fibers on the seed surface, which is different from *G. hirsutum* varieties whose seed surface are usually covered with a high density of fuzz fibers. It is often observed that the fuzz density (FD) of the same *G. barbadense* variety varies with the environmental context, and the FD in Aksu (Xinjiang, China) and Sanya (Hainan, China) is lower than that in Anyang City (Henan, China) [35]. In this study, 121 *G. barbadense* varieties were used for FD evaluation. The temperature in the three cultivation cities (Sanya, Hainan; Anyang, Henan; Korla, Xinjiang) during the florescence period was investigated. Considering the FD, variety L7009 was the most sensitive to environmental stimuli. We also verified the influence of temperature on fuzz initiation of L7009 and the time when fuzz initiation occurred and examined the fiber quality at different temperatures. To understand the transcriptional regulation mechanism of temperature on the fuzz initiation and the early development of fibers, we conducted comparative transcriptome on ovules of L7009 in different temperature environments. Then we performed functional enrichment analysis and co-expression analysis of differentially expressed genes from major profiles. These findings in this study extend our understanding of how temperature affects the development of cotton fibers and provide valuable data for studying the mechanism of temperature-regulated fuzz fiber development.

## 2. Materials and Methods

### 2.1. Plant Materials and Temperature Treatments

A total of 121 *G. barbadense* varieties (provided by the Cotton Research Institute of the Chinese Academy of Agricultural Sciences) were used to investigate the effects of different environments (Anyang, Sanya, and Korla) on fuzz density and lint quality. A variety named L7009 with dense stem and leaf trichomes [36] was used to evaluate the impact of temperature on fuzz density and fiber quality. We simulated the high temperature (HT) environment with 36 °C in the day and 28 °C at night and the low temperature (LT) environment with 25 °C in the day and 20 °C at night. Both environments had the same photoperiod (15 h light/9 h dark) and light intensity. Firstly, we transplanted the three-leaf seedlings into flowerpots with one plant per pot and then transferred them to the greenhouse. When it came to the full-bloom stage, 120 cotton plants were equally divided into two groups and transferred into HT and LT climatic chambers, respectively. The flowers were artificially pollinated every day to ensure the fruit setting rate. When transferred into the cotton climatic chambers on the fifteenth day, ovules of 0 to 7 dpa in the two environments were separately collected and stored in liquid nitrogen. Each biological replicate at each stage came from a mixed ovule sample of three bolls. The mature seeds were harvested bolls were split and dried. The seeds and lint fibers were manually separated, fuzz density was investigated, and fiber quality was measured. To reveal when fuzz initiation occurred, we planted another batch of L7009 plants, and the management was the same as the previous one. To accurately track the temperature changes sensed by each seed during fiber development, sixty plants in each environment were numbered, and dated cards were hanged on the flowers every day. After ten days, cotton plants from two chambers were exchanged. The flowers bloomed on the day of transfer environment were recorded as 0 days after anthesis when transferred (DAT), those bloomed on the previous day were recorded as 1 DAT, and those bloomed on the second day after the exchange were recorded as −1 DAT. Flowers that bloomed every day were tagged with dated cards.

### 2.2. Ovule Samples Preparation for SEM Analysis

Ovule samples collected from HT and LT environments were immersed in an electron microscope fixative and vacuumed. The fixed samples were rinsed with 0.1 M phosphate buffer PB (PH 7.4) three times. The samples were fixed with 1% osmium and 0.1 M phosphate buffer PB (PH 7.4) at room temperature (20 °C) for 1~2 h and then rinsed with 0.1 M phosphate buffer PB (PH 7.4) for three times. The samples were successively dehydrated with 30% to 100% alcohol solution for 15-min intervals, and then dehydrated with isoamyl acetate for 15 min. After critical point drying and ion sputtering, the sample was observed by scanning electron microscope (SEM).

### 2.3. Fuzz Phenotype Determination and Fiber Quality Measurement

The fuzz density of mature seeds was classified into eight grades by visual inspection. The seeds scored “0” were fuzzless, and those with a score of “3.5” were covered with dense fuzz fibers. The fuzz density score was used to characterize the fuzz phenotype of the seeds quantitatively, and it was measured in the same way as previously reported [37]. After the fibers and seeds were separated manually, the seed size (SS), fuzz density (FD), and the hundred-grain weight (HGW) were determined, respectively. The lint fiber samples were sent to the Center of Cotton Fibre Quality Inspection and Testing, Chinese Ministry of Agriculture (Anyang, Henan province, China) for fiber quality testing, including High Volume Instrument (HVI) analysis and Advanced Fiber Information System (AFIS) analysis.

### 2.4. Transcriptome Sequencing and Data Analysis

Ovules of 1, 4, and 7 dpa were collected from HT and LT and conserved in liquid nitrogen, with two biological replicates per treatment. The RNAprep Pure Plant Plus Kit (No. DP441; Polysaccharides and Polyphenolics-rich) (TIANGEN, Beijing, China) was then used to extract the total RNA according to its standard procedure. RNA purity was measured with a NanoDrop 2000 microspectrophotometer, its concentration was measured with an Agilent 2100 Bioanalyzer, and integrity was monitored with an Agilent RNA 6000 Nano Kit (Agilent Technologies, Inc., Beijing, China). The cDNA libraries construction and transcriptome sequencing were completed by Annoroad Gene Technology Co., Ltd. (Beijing, China). The company used the PE150 sequencing method of the Illumina HiSeq 4000 sequencing platform to complete the sequencing project and feed raw data back to us for further analysis. The clean data were obtained from the raw reads after removing the adaptor sequences and low-quality reads. High-quality sequences were aligned with the latest version genome of *G. barbadense*, which can be available at http://ibi.zju.edu.cn/cotton. The FPKM (fragment per kilobase of transcript per million mapped reads) value of each gene in each sample was obtained by Cufflinks software (http://cole-trapnell-lab.github.io/cufflinks/) (Laboratory for Mathematical and Computational Biology at UC Berkeley, Institute of Genetic Medicine at Johns Hopkins University, and Barbara Wold’s lab at Caltech, USA). Genes with FPKM < 0 were removed, and genes with FPKM ≥ 1 in at least one sample were defined as expressed genes. Pearson correlation coefficient analysis and principal component analysis were performed with all FPKM > 0 genes. The edgeR package [38] of R v. 3.6.2 software (Bell Laboratories, Madison, WI, USA) was used to analyze the differentially expressed genes, and the genes with |Log_2_FC| ≥ 1 and *p*-value < 0.01 were assigned as differentially expressed. The Mfuzz program [39] of R v. 3.6.2 software was used for clustering analysis. Gene ontology (GO) and Kyoto Encyclopedia of Genes and Genomes (KEGG) databases (https://www.omicshare.com/tools/) were used for functional annotation and pathway enrichment analysis of genes in important modules. The interaction relationship of genes with membership ≥ 0.70 in each module was obtained from the STRING database (https://string-db.org/). The interaction relationship was imported into Gephi v. 0.9.2 software (https://gephi.org/) to construct a co-expression network diagram.

### 2.5. Determination of Physiological and Biochemical Phenotype

Data analysis showed that some genes potentially related to fuzz fiber initiation were involved in stress response and ROS homeostasis, so the activities of superoxide dismutase (SOD), peroxidase (POD), catalase (CAT), and ascorbate peroxidase (APX) were determined, and the contents of H_2_O_2_ and oxygen-free radical (OFR) were also measured. L7009 ovules were collected from HT and LT environments at three time points based on RNA-seq sampling time and frozen in liquid nitrogen. The six physiological and biochemical indexes were determined according to the manufacturer’s protocol of assay kits (http://www.cominbio.com/, kit NO. H2O2-1-Y for H2O2; kit NO. SA-1-G for OFR; kit NO. POD-1-Y for POD activity; kit NO. SOD-1-W for SOD activity; kit NO. CAT-1-Y for CAT activity; kit NO. APX-1-W for APX activity) (Comin Biotech, Suzhou, China).

### 2.6. Validation of RNA-Seq Data by qRT-PCR

Total RNA was extracted with RNAprep Pure Plant Plus Kit (TIANGEN Biotech Co., Ltd., Beijing, China). The first strand of cDNA was synthesized using RNA and the PrimeScript^TM^ II 1st Strand cDNA Synthesis Kit. All qRT-PCR primers were designed using Oligo 7 software [40] and synthesized by Sangon Biotech Co., Ltd. (Shanghai, China). The *GbUBQ7* was used as a reference gene [41]. Follow the system and procedures recommended by the UltraSYBR Mixture (Beijing ComWin Biotech Co., Ltd., Beijing, China) instructions. qRT-PCR was performed on a 7500 real-time PCR system. The relative expression of genes was analyzed by the 2^−ΔΔCT^ method [42]. The experiment was performed in three biological replicates, with three technical replicates in each biological replicate.

## 3. Results

### 3.1. Environmental Factors Affect the Fiber Properties of G. barbadense

**Figure 1:**
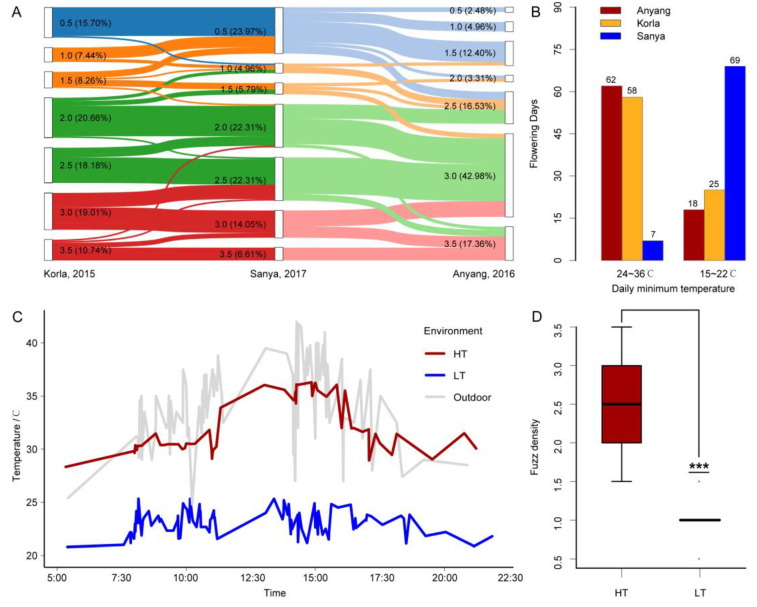
Dynamic change of fuzz density and verification of the environmental factor. (**A**) The change of fuzz density between the three natural environments (Korla, Anyang, and Sanya). The fiber density is divided into seven grades, and the percentage of varieties corresponding to each grade is displayed. The width of the strip indicates the number of varieties. (**B**) Statistics of daily minimum temperature during the three-year flowering period in the three natural environments. The daily minimum temperature is divided into two groups of 24~36 °C and 15~22 °C, representing the number of high-temperature days and low-temperature days, respectively. (**C**) Real-time temperature monitoring of artificial climate chamber. (**D**) Fuzz density in high temperature (HT) and low temperature (LT) environments. The number of seeds investigated in HT and LT was *n* = 64 and *n* = 57. The number of asterisks represents a significant level (***, *p* < 0.001).

To reveal the environmental influence on fuzz and lint fibers development, we investigated fuzz density (FD) and lint fiber quality of 121 *G. barbadense* cultivars in three cities (Korla, Anyang, and Sanya) (Table S1). For convenience, we visually graded fuzz density to eight levels (Figure S1). In Sanya, 23.97% of the cultivars showed low fuzz density (FD = 0.5) and 20.66% showed high fuzz density (FD = 3~3.5). However, in Korla and Anyang, 15.70% and 2.48% of the cultivars showed low fuzz density, while 29.75% and 60.34% showed high fuzz density, respectively (Figure 1A). This result suggests that the environment of Sanya is more likely to reduce fuzz density, and the environment of Anyang is more likely to increase fuzz density. We also investigated the relationship between lint quality and fuzz density of 98 varieties planted in Sanya. Three of five fiber quality attributes, fiber length (FL), fiber strength (FS) and fiber elongation (FE), showed a significant positive correlation with fuzz density (FL, *r* = 0.59; FS, *r* = 0.61; FE, *r* = 0.47) (Figure S2). Recalling that temperature could affect fiber quality [43], we investigated the daily minimum temperature of three environments during the flowering period of three years (2015, 2016, and 2017). The days with daily minimum temperature above 24 °C accounted for 64.6% of the total survey days (n = 96) in Anyang, while 71.9% of the days (n = 93) were below 22 °C in Sanya (Figure 1B). Therefore, we conjecture that temperature may be a key environmental factor affecting the fuzz density of *G. barbadense*, and high temperatures may increase the fuzz density.

### 3.2. Temperature Regulates Fuzz Fiber Initiation and Affects Lint Fiber Development

**Figure 2:**
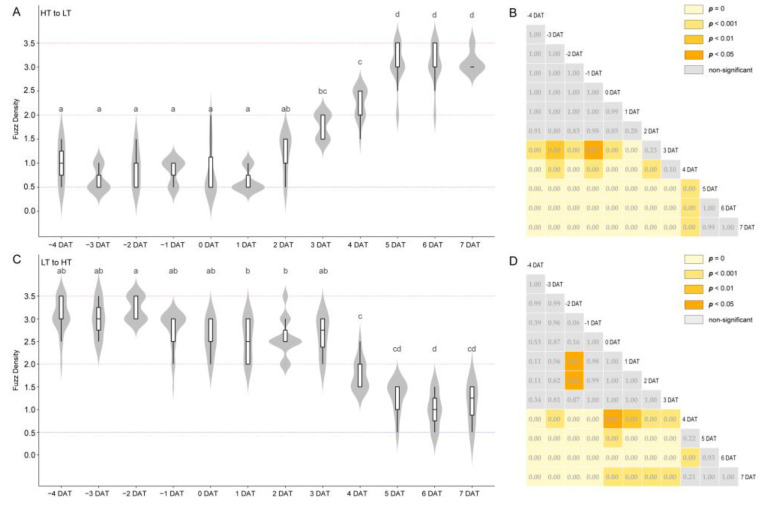
Verification of the fuzz initiation period of island cotton. After being moved from HT to LT, the dynamic variation of fuzz density with time (**A**) and the significant difference of fuzz density between different treatment depths (**B**). After being moved from LT to HT, the dynamic variation of fuzz density with time (**C**) and the significant difference (*p*-value) matrix of fuzz density between different treatment depths (**D**). Different letters (a–d) above each set of box and whiskers indicate significantly different at *p* < 0.05. Tukey’s HSD statistical test was used for pairwise comparison.

**Figure 3:**
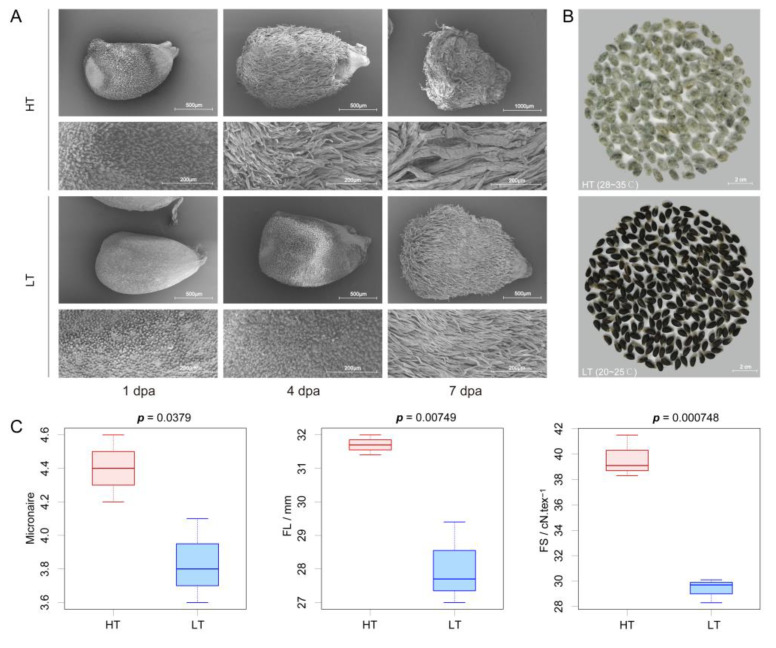
Impact evaluation of different temperatures on developing ovules, fuzz density, and fiber quality. (**A**) Scanning electron microscopy of ovules and corresponding epidermal fibers affected by high and low temperatures during fuzz fiber development. Scale bar: 500 μm (the first two columns of the first row and the third row); 1 mm (the third column of the first row); 200 μm (the second and fourth rows). Rows two and four are enlarged versions of the SEM images on rows one and three, respectively. (**B**) Mature seeds developed in high (Temp > 28 °C, *n* = 191) and low (Temp < 25 °C, *n* = 311) temperature environments. (**C**) The fiber quality of fiber length (FL), fiber strength (FS), and Micronaire developed under high and low-temperature environments measured using a High Volume Instrument (HVI) system.

To test our hypothesis, we simulated the ambient temperature of the natural environments (Figure 1C). Fortunately, we discovered one island cotton variety L7009 with different fuzz density in Anyang and Sanya (Anyang, FD = 3.5; Sanya, FD = 0.5), which was not planted in Korla and was not included in the 121 varieties mentioned above. The fuzz density difference of L7009 in Anyang and Sanya was more significant than that of other varieties, so we selected L7009 for further study. We found that the fuzz density of L7009 in high temperatures (HT, 28~35 °C) was significantly higher than that in low temperatures (LT, 20~25 °C), while other external factors in both environments were the same, such as photoperiod, light intensity, water and fertilizer (Figure 1D). This result indicates that temperature is the decisive environmental factor regulating fuzz initiation of L7009 and that high temperature can increase fuzz density. Based on the fact that the fuzz density of L7009 was significantly affected by temperature, we designed an experiment to confirm the fuzz initiation period and the day most susceptible to temperature. First, we transferred a batch of cotton plants at the full flowering stage from HT to LT (HTL). The flowers bloomed on the day of transfer were recorded as 0 days after anthesis when transferred (DAT), those bloomed on the previous day were recorded as 1 DAT, and those bloomed on the second day after transfer were recorded as −1 DAT. We found that the fuzz density at 4 DAT was significantly lower than that at 5 DAT or higher than that at 2 DAT, while there was no significant difference when compared to 3 DAT, indicating that 3~4 dpa was a crucial period for fuzz initiation development (Figure 2A,B). To make the results more credible, we transported another batch of cotton plants from LT to HT (LTH), and found that the fuzz density at 4 DAT was significantly lower than that at 3 DAT or higher than that at 6 DAT, while there was no significant difference when compared to 5 DAT, indicating that 4~5 dpa was also important for fuzz initiation (Figure 2C,D). By comparing the fuzz density of HTL and LTH at the same stages, it was found that there was no significant difference between HTL and LTH at 4 dpa. In contrast, significant differences between any other stages were found, indicating that 4 dpa was the critical stage for fuzz initiation development, which was consistent with previous findings in *G. hirsutum* (Table S2) [4]. The experiments could also prove that 3~5 dpa was related to the fuzz initiation development of island cotton. The temperature could not significantly affect the fuzz density before 2 dpa or after 6 dpa, which indicated that the fuzz fiber initiation had not started before 2 dpa, but had ended after 6 dpa. More conservatively, we cautiously designate 1 dpa and 7 dpa as the stages before and after the development of fuzz differentiation, respectively. With phenotypic observation, we found that high temperatures could accelerate lint fiber initiation and growth and increase the fuzz density on the seed surface (Figure 3A,B). In addition, it was found that short fiber content based on the number (SFC_n), fiber strength (FS), 100-grain weight (HGW), fuzz percentage (FP), seed size (SS), length based on weight (Length_w), upper quartile length (UQL), mature ratio (MR), fiber neps content (FNC), seed coat neps content (SCNC), fiber length (FL), short fiber content based on weight (SFC_w), Fineness, seed coat neps mean size (SCNMS) and Micronaire of L7009 in HT were significantly (*p* < 0.05) different from those in LT (Figure S3 and Figure 3C). Among them, six fiber attributes in HT were markedly better than those under LT, and the differences in length based on the number (Length_n) and immature fiber content (IFC) were not significant. These results showed that temperature difference could regulate the fuzz density of L7009, and significantly affect the development and quality of lint fibers.

### 3.3. Transcriptome Sequencing and Comparative Analysis of Differentially Expressed Genes between Different Stages and Environments

To reveal the molecular regulation mechanism of ambient temperature on the fuzz initiation development of L7009, we constructed cDNA libraries from 12 samples of three stages (1, 4, and 7 dpa) under two environments (HT and LT). The cDNA libraries were then sequenced using an Illumina HiSeq 4000 sequencing platform based on paired-end sequencing. All RNA-seq raw datasets were deposited in the NCBI database with an SRA accession number PRJNA598978. In total, we obtained 73.43 Gb of clean data after mRNA sequencing for 12 samples with at least 6.00 Gb of clean data for each sample. In each sample, more than 93.30% of bases score Q30 and above (Table 1). The clean data were then mapped to the reference genome of *G. barbadense* (http://ibi.zju.edu.cn/cotton), with the mapping ratio varying from 90.62% to 95.17%. Based on the alignment results, alternative splicing predictions, genetic structural optimization, and novel gene discovery were performed. Finally, 5994 novel genes were identified, 5180 of which were functionally annotated (Table S3). Based on the alignment results, gene expression level analysis was performed. We utilized Cufflinks software to estimate its expression level based on a maximum flow algorithm and used FPKM (Fragments Per Kilobase of transcript per Million fragments mapped) value to measure the transcript expression level. A total of 63,113 transcripts were obtained with FPKM values > 0 in at least one sample, of which 44,864 were expressed with FPKM values ≥ 1.

**Figure 4:**
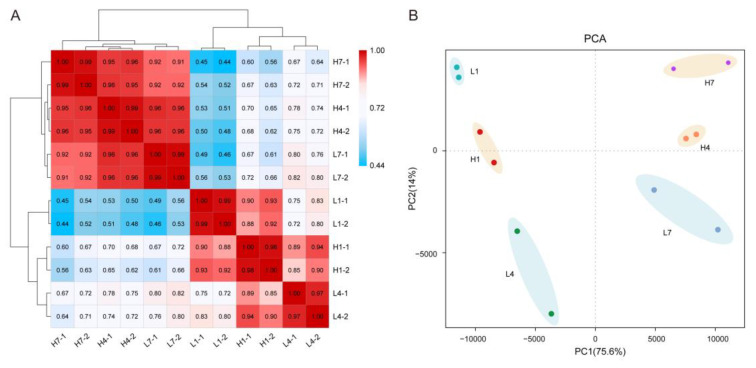
Relationship between transcriptome samples. (**A**) Cluster dendrogram and Pearson correlation coefficient heatmap based on normalized FPKM (fragment per kilobase of transcript per million mapped reads) values of expressed genes. (**B**) PCA analysis of transcriptome data from twelve samples. The dots in the bisque background represent samples from the HT environment, and the dots in the powder blue background represent samples from the LT environment.

The Pearson correlation coefficient between two biologically repeated samples of the same stage in different environments fluctuated between 0.98 and 0.99. The sample clustering also showed a good correlation between the two biological replicates (Figure 4A). The results of principal component analysis (PCA) of the twelve samples were consistent with the above results (Figure 4B).

Intra- and inter-environment comparisons of all expressed genes were performed to reveal the dynamic transcriptional changes during fuzz initiation. In total, 26,048 differentially expressed genes (DEGs) were identified under the thresholds of absolute log_2_ ratio ≥ 1.0 and *p*-value < 0.01. In HT, 14,490 DEGs were found at 1 dpa vs. 4 dpa (3393 up-regulated, 11,097 down-regulated), 2160 DEGs at 4 dpa vs. 7 dpa (1230 up-regulated, 930 down-regulated), and 12,716 DEGs at 1 dpa vs. 7 dpa (3628 up-regulated, 9088 down-regulated) (Table 2 and Figure S4A). In LT, 5694 DEGs were found at 1 dpa vs 4 dpa (1473 up-regulated, 4221 down-regulated), 4290 DEGs at 4 dpa vs. 7 dpa (1667 up-regulated, 2623 down-regulated), and 17,748 DEGs at 1 dpa vs. 7 dpa (3352 up-regulated, 14,396 down-regulated) (Table 2 and Figure S4A). Compared to LT, the HT samples had 2480 DEGs at 1 dpa (951 up-regulated, 1529 down-regulated), 7355 DEGs at 4 dpa (3072 up-regulated, 4283 down-regulated), and 4164 DEGs at 7 dpa (2876 up-regulated, 1288 down-regulated) (Table 2 and Figure S4A). Of these DEGs, the genes differentially expressed between LT and HT treatments at the same stages were identified as temperature-responsive. Those identified at different stages in the same temperature environment were defined as possibly related to fiber development. Therefore, we identified 9667 DEGs that were likely to be involved in temperature response and fiber development (Figure S4B).

### 3.4. Gene Expression Trend Analysis and Protein-Protein Interaction (PPI) Network Construction

**Figure 5:**
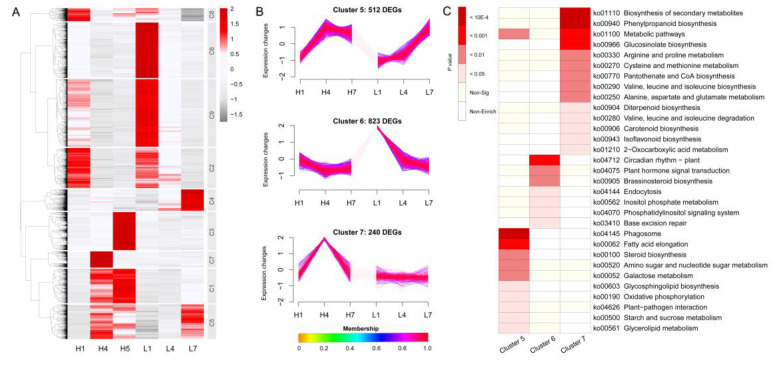
Hierarchical cluster analysis of differentially expressed genes (DEGs). (**A**) Heatmap of the expression profiles of 9667 DEGs participating in both temperature response and fiber development. The color scale represents the Z-score of the gene expression level. (**B**) Expression trends of genes in the three clusters related to fiber and fuzz development. (**C**) KEGG (Kyoto Encyclopedia of Genes and Genomes) pathway enrichment analysis of the three clusters related to fiber development. The color scale on the left of the heatmap represents different levels of significance.

The 9667 DEGs were subjected to hierarchical clustering using the Mfuzz package of R v. 3.6.2 software and divided into nine clusters (Figure S5), in which 4756 (49.2%) genes with membership value ≥ 0.7 were illustrated with a hierarchical clustering heatmap (Figure 5A). The expression levels of 512 genes in cluster 5 were continuously up-regulated from H1 to H7 and L4 to L7 (Figure 5B). Genes in clusters 6 (823) and 7 (240) were predominantly expressed at L1 and H4, respectively (Figure 5B). The previous experimental results showed that at 4 dpa, the fuzz initiation was significantly induced by high temperatures, but inhibited by low temperatures. Among all the gene co-expression modules, the genes in cluster 7 were only up-regulated in H4, but not induced in low temperatures or with a very low expression level, indicating that these genes might be involved in fuzz initiation induced by high temperatures. Combined with phenotypic observation, genes in cluster 5 and 6 were likely to be respectively involved in lint fiber elongation and initiation under temperature regulation. However, most of the genes were enriched in clusters 5 and 6, and only a small number of genes were recruited into cluster 7, which indicated that the mechanism of lint fiber development affected by temperature was very complicated.

**Figure 6:**
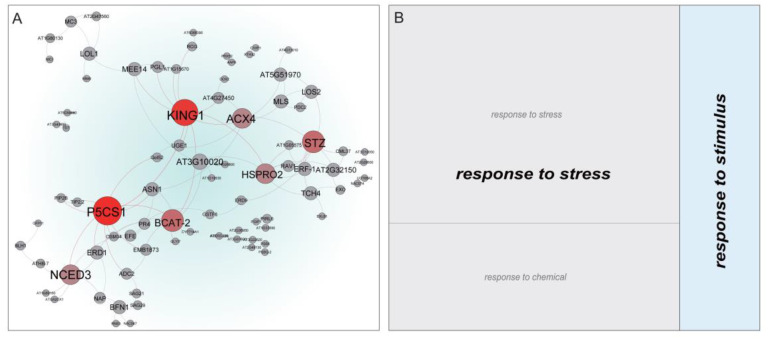
PPI (Protein-Protein Interaction) network and potential functions of the genes in cluster 7. (**A**) PPI network based on co-expressed genes. The larger the node, the more genes that interact with it in the module. The nodes with red gradient colors represent the hub genes, and their names are highlighted in bold. (**B**) Treemap of BP (biological process) gene ontology (GO)-terms found in the PPI network. Each rectangle represents a single cluster of related terms. Related GO-terms were clustered together in a supercluster of the same color. The size of each rectangle was adjusted to reflect the *p*-value of the enriched GO-terms.

The 240 genes with membership value ≥ 0.7 in cluster 7 were mapped to the STRING database (https://string-db.org/). The PPI (Protein-Protein Interaction) network was visualized using the Gephi v. 0.9.2 software to investigate the possible interaction between the differentially expressed genes (Figure 6A). In the PPI network, the node genes with larger degrees were defined as hub genes. In the cluster 7, *P5CS1* (*GB_D01G2569*), *KING1* (*GB_A12G2167*), *STZ* (*GB_D05G2149*), *BCAT-2* (*GB_A05G2168*), *ACX4* (*GB_A13G1045*), and *HSPRO2* (*GB_A10G2743*) were identified as hub genes, which were considered as potential candidate genes involved in regulating fuzz initiation induced by high temperatures.

### 3.5. Gene Ontology (GO) and KEGG Pathway Analysis of DEGs

There is a partial overlap in the developmental stages of lint elongation and fuzz initiation. To further understand the DEGs functions in the three clusters, GO-term enrichment for each cluster was performed (Table S4). To reduce the functional redundancy among GO-terms, we used the REVIGO program (http://revigo.irb.hr/) and the treemap package (https://CRAN.R-project.org/package=treemap) of R v. 3.6.2 software to obtain and visualize overrepresented GO-terms [44].

**Figure 7:**
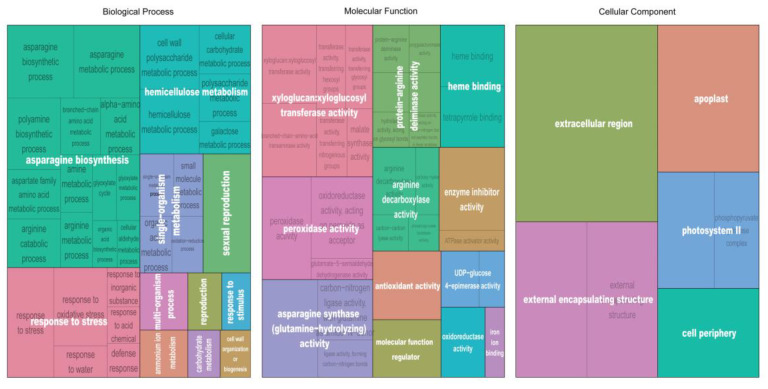
Treemap of GO-terms found in cluster 7. Each rectangle represents a significant GO-term. Related GO-terms were clustered together in a supercluster of the same color, and all supercluster names were labeled in white. The sizes of rectangles were adjusted to reflect the relative corrected *p*-value.

The DEGs in cluster 5 were significantly (FDR < 0.05) enriched into 30 GO-terms of biological process, of which the terms involved “fatty acid biosynthetic process”, “monocarboxylic acid biosynthetic process”, “lipid biosynthetic process”, “microtubule-based process”, and “intracellular protein transport” were the most significant (Table S4). Forty significant terms for molecular functions were enriched, and the most significant of which were redundant and involved in “structural constituent of cytoskeleton”, “GTPase binding”, “UDP-glycosyltransferase activity”, and “actin binding” (Table S4; Figure S6). For the cell component, 16 terms were enriched, and the most significant involved “microtubule”, “cytoskeleton”, and “membrane” (Table S4). The DEGs in cluster 6 were significantly enriched into 104 GO-terms of biological process, of which the supercluster terms involved “glycerolipid metabolism”, “dephosphorylation”, and “protein deacetylation” (Figure S6). Fifty-one significant terms for molecular functions were enriched, the superclusters of which involved “phosphoric ester hydrolase activity”, and “1-phosphatidylinositol-4-phosphate 5-kinase activity” (Figure S6). For the cell component, 10 GO-terms were significantly enriched, and “cytosol” was the main supercluster (Figure S6). The DEGs in cluster 7 were significantly (*p* < 0.05) enriched into 63 GO-terms of biological process, of which the supercluster terms involved “asparagine biosynthesis”, “response to stress”, and “hemicellulose metabolism” (Figure 7). Forty-one significant terms for molecular functions were enriched, the superclusters of which involved “xyloglucan:xyloglucosyl transferase activity” and “peroxidase activity” (Figure 7). Nine significant terms for cell components were enriched, and the supercluster terms of treemap involved “extracellular region” and “external encapsulating structure” (Figure 7). Genes in the PPI network of cluster 7 were mainly enriched in the GO-term superclusters “response to stress” and “response to stimulus” (Figure 6B).

At the same time, we performed KEGG pathway enrichment analysis on the three clusters (Table S5). DEGs in clusters 5, 6, and 7 were significantly (*p* < 0.05) mapped into 11, 7, and 14 KEGG pathways, respectively, suggesting that there might be more pathways involved in fuzz initiation than early lint fiber development in high temperatures. The most significant pathways in cluster 5 were “phagosome” (14 DEGs), “fatty acid elongation” (5 DEGs), “metabolic pathways” (59 DEGs), and “amino sugar and nucleotide sugar metabolism” (9 DEGs) (Figure 5C). The most significant pathways in cluster 6 were “circadian rhythm” (7 DEGs), “plant hormone signal transduction” (18 DEGs), and “brassinosteroid biosynthesis” (3 DEGs) (Figure 5C). And the top three pathways with most DEGs in cluster 7 were “biosynthesis of secondary metabolites” (29 DEGs), “phenylpropanoid biosynthesis” (8 DEGs), and “metabolic pathways” (32 DEGs) (Figure 5C).

### 3.6. Differentially Expressed Transcription Factors Involved in Fuzz Fiber Initiation

Numerous studies have shown that the development of *Arabidopsis* trichome or cotton fiber was strictly regulated by several transcription factors [45,46,47,48,49,50]. To identify TFs associated with fuzz initiation, we detected twenty-five differentially expressed TF genes assigned to thirteen families in cluster 7. Totally, 20 and 105 TF genes were identified in clusters 5 and 6, respectively (Table S6). Of these, there were more genes involved in lint fiber initiation, and relatively few genes involved in fuzz fiber initiation. Different genes of four TF families, such as NAC, bHLH, MYB, and C2H2, were discovered in the three important clusters. However, transcription factors of four families, such as AP2/ERF (ERF), C2C2-LSD, HD-ZIP, and PLATZ, were mainly identified in clusters 6 and 7. In addition, six genes distributed in five families (*RAV1*, *BLH1*, *HB40*, *LBD1,* and *OFP11*) were exclusively enriched in cluster 7. According to the PPI network, the *STZ* gene encoding a Cys2/His2-type zinc-finger protein was identified as the key transcription factor of cluster 7, which might play a pivotal role in fuzz initiation induced by high temperatures.

### 3.7. DEGs Involved in Phytohormone Signal Transduction Pathway

Previous studies have found that cotton fiber development can be regulated by genes related to plant hormone signal transduction. We identified 189 genes related to hormone signal transduction from the 9667 DEGs involved in temperature response and fiber development. Eighteen unigenes were from cluster 6, including 7 Auxin (*AUX1*, *IAA,* and *SAUR*), 5 Abscisic acid (*PYR*/*PYL*, *PP2C*, *SnRK2,* and *ABF*), 3 Cytokinine (*CRE1*, *AHP,* and *B-ARR*), 1 Ethylene (*CTR1*), and 2 Brassinosteroid (*CYCD3*) signal transduction component-encoding genes (Table S6). Five unigenes were from cluster 7, and these genes encode the components of Auxin (*GH3*) and Brassinosteroid (*TCH4*) signal transduction, respectively (Table S7). There were more genes in cluster 6 than that in cluster 7. For example, no genes related to abscisic acid, cytokinin, and ethylene signal transduction were found in cluster 7, but several genes were identified in cluster 6. Although some genes for the auxin and brassinosteroid signaling pathways were found in both cluster 6 and cluster 7, the components encoded by these genes were different, indicating that *GH3* and *TCH4* may play a role in regulating fuzz initiation induced by high temperatures.

### 3.8. Transcriptional Changes of Genes Related to Cotton Fiber Initiation

**Figure 8:**
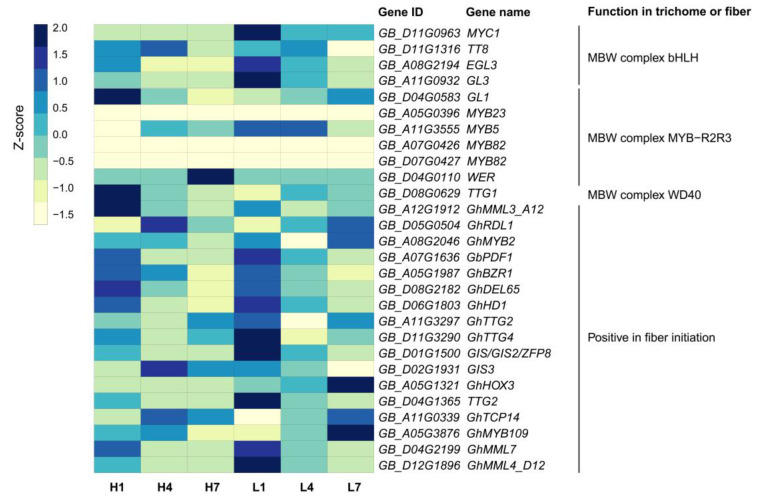
Heatmap of the genes potentially related to fiber initiation.

It is believed that the homologous genes related to the trichome initiation of *Arabidopsis* may also be involved in the cotton fiber initiation [51]. According to previous reports [26,52,53], We identified 28 homologous genes related to the trichome initiation of *Arabidopsis* and fiber initiation of cotton from the *G. barbadense* genome and found that they had different expression patterns in developing ovules under different temperatures (Figure 8). Obviously, most genes were significantly up-regulated in L1, such as *MYC1* (*GB_D11G0963*), *EGL3* (*GB_A08G2194*), *GL3* (*GB_A11G0932*), *GbPDF1* (*GB_A07G1636*), *GhHD1* (*GB_D06G1803*), *GhTTG4* (*GB_D11G3290*), *GIS* (*GB_D01G1500*), *TTG2* (*GB_D04G1365*), *GhMML7*/*GhMYB25* (*GB_D04G2199*), and *GhMML4_D12* (*GB_D12G1896*). Four genes, including *GL1* (*GB_D04G0583*), *TTG1* (*GB_D08G0629*), *GhMML3_A12*/*GhMYB25-like* (*GB_A12G1912*) and *GhDEL65* (*GB_D082182*), were significantly up-regulated in H1. The nineteen genes were mainly expressed in the initiation stage of lint fibers (1 dpa), which indicated that they were potentially related to lint initiation development. The four genes *TT8* (*GB_D11G1316*), *GhRDL1* (*GB_D05G0504*), *GIS3* (*GB_D02G1931*), and *GhTCP14* (*GB_A11G0339*) have higher expression levels in H4, but their expression levels were lower in low temperatures, suggesting that they were potentially related to fuzz initiation development. No gene was predominantly expressed in L4, which might partly explain why fuzz fibers didn’t initiate at 4 dpa in LT. Two genes related to fiber elongation development, *GhHOX3* (*GB_A05G1321*) and *GhMYB109* (*GB_A05G3876*), were up-regulated in L7. Interestingly, *MYB23* (*GB_A05G0396*) and *MYB82* (*GB_A07G0426* and *GB_D07G0427*) were not expressed in all samples.

### 3.9. Changes in Antioxidant Enzyme Activity and ROS Content during Fuzz Initiation

**Figure 9:**
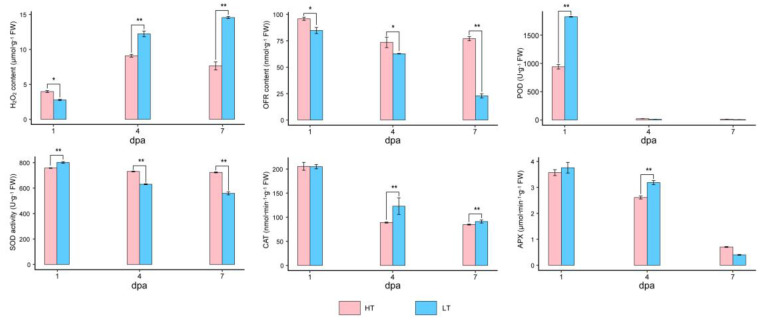
Antioxidant enzyme activities and ROS levels under different temperature environments during early fiber development. The height of the bar represents the mean value, and the error bar represents mean ± SE. The number of asterisks represents a significant level (*, *p* < 0.01; **, *p* < 0.01).

According to the GO-term enrichment analysis, some genes in cluster 7, such as *RCI3* (*GB_A03G1340*), *DOX2* (*GB_A09G2023*), *PRX52* (*GB_A03G0328*), *GPX4* (*GB_D08G0779*) and *ERD10* (*GB_A01G1053*), involved in stress response were potentially related to fuzz initiation induced by high temperatures. Therefore, we measured the activities of four antioxidant enzymes and the contents of H_2_O_2_ and oxygen-free radical (OFR) (Figure 9). The results showed that the H_2_O_2_ content at 4 dpa was significantly higher than 1 or 7 dpa in the HT environment, and the content at 4 dpa in the LT environment was substantially higher than that of the HT environment. The content of OFR in the early development stage of fibers in the HT environment was generally higher than that in LT environment, indicating that the two ROS, OFR, and H_2_O_2_, might play different roles in fuzz initiation development. The four enzymes activity showed a downward trend from 1 to 7 dpa in different temperature environments. Among them, the activities of SOD, CAT, and APX at 4 dpa in the HT environment were generally higher than those in the LT environment. This indicates that the regulation level of ROS homeostasis is an important cause of the difference during fuzz fiber initiation at different temperatures.

### 3.10. Verification of RNA Sequencing Data by qRT-PCR

**Figure 10:**
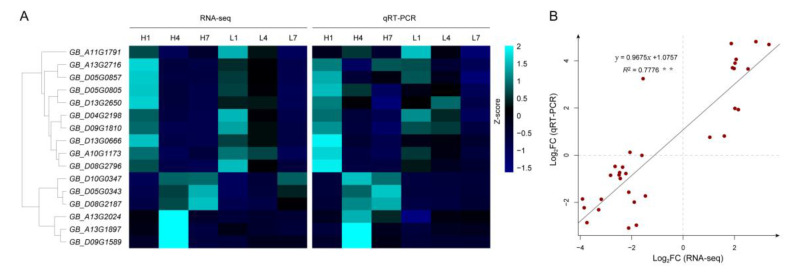
Validation of the RNA-seq data by qRT-PCR. (**A**) Heat map of RNA-seq data and qRT-PCR data of sixteen genes randomly selected. (**B**) Correlation between RNA-seq data (FPKM) and qRT-PCR results (2^−ΔΔCt^) of the sixteen genes. Scatter points represent the fold-changes in expression levels at 4 dpa compared with 1 dpa. The *R*^2^ value represents the correlation between RNA-seq data and qRT-PCR results.

To validate the RNA-seq data, we performed quantitative real-time PCR (qRT-PCR) for sixteen genes randomly selected from different expression profiles. The sixteen genes and their primer sequences were listed in Table S8. In general, the relative expression levels based on qRT-PCR were consistent with the results of RNA-seq, and the correlation between them was significant (*R*^2^ = 0.78) (Figure S7 and Figure 10B). However, when we clustered the sixteen selected genes according to the RNA-seq data and qRT-PCR results, we found that *GB_A01G1791* was not clustered with the other three genes of cluster 7, but with the genes of cluster 2 and 9 (Figure 10A). We then calculated the correlation between the nine clusters based on the eigengenes and found that the Pearson correlation of cluster 2 and cluster 9 reached 0.94, indicating that the gene expression patterns from these two clusters were similar (Table S9). These results showed that the RNA-seq data was credible and accurate.

## 4. Discussion

### 4.1. Temperature is the Key External Factor that Determines the Fate of Epidermal Cells of G. barbadense Ovules

As an important fiber source plant, *G. barbadense* has been favored by scientific researchers and textile industries for its excellent fiber quality attributes. The quality of cotton fibers is sensitively affected by environmental stimulus [54,55]. The difference in fiber quality mainly comes from meteorological factors (e.g., temperature and rainfall) and soil factors [56]. With sufficient water and fertilizer supply, light and temperature are the most likely environmental factors affecting fiber development. However, light cannot be directly perceived by the ovules like temperature. When simulating the winter temperature in Sanya and the summer photoperiod in Anyang, it was found that long daylight could not effectively induce fuzz fiber initiation compared with the environment in Sanya (short day). We also simulated the summer temperatures and photoperiod of Anyang, and found that the low light intensity of the cultivation room could not reduce the fuzz density when compared with the natural environment (high light intensity). All these results suggest that light is not the decisive environmental factor for the fuzz fiber initiation of L7009. Of these environmental stimuli, temperature is the most crucial factor affecting fiber quality. Low temperature stress, especially the mean daily minimum temperature (<20 °C), is the major abiotic stress limiting the formation of cotton fiber quality, such as fiber length [43]. In this study, it led to significant differences in fiber quality when the ambient temperature was set to 20~25 °C and 28~36 °C. Interestingly, high temperatures significantly increased the fuzz density (FD) when compared to a low temperature. These findings indicate that the change of FD is potentially regulated by ambient temperatures. Moreover, with the increase of temperature, the quality of fiber length (FL), fiber strength (FS), and fiber elongation (FE) were also improved accordingly, indicating that fiber quality was also affected by ambient temperature. However, the underlying mechanism remains unknown, and the relevancy between FD and lint fiber quality attributes is noticeable. In our study, it was found that the elevated temperature could indeed accelerate the early fiber elongation process (Figure 3A). Nevertheless, if the temperature is too high, it will shorten the fiber rapid elongation duration and terminate the elongation progress earlier, leading to the reduction of final fiber length [57]. On the contrary, the relatively low temperatures can also encumber fiber initiation and elongation process [58].

Utilizing scanning electron microscopy and fiber mutants of *G. hirsutum*, it had been found that fuzz initiation occurred at 4 dpa [4]. We also found that the fuzz initiation of another allotetraploid cotton species, *G. barbadense*, also mainly occurred around 4 dpa. This suggests that the two allotetraploid cotton species are identical in the fuzz fiber initiation time. Based on the quantitative trait locus (QTL) mapping method, some fuzzless loci have been reported, including *N*_1_ and *n*_2_. The homozygous *N*_1_*N*_1_ is completely fuzzless, and the fuzz development of the *n*_2_ mutant is reported to be controlled by genetic background and environmental conditions together [59,60]. Most *G. barbadense* varieties contain the *n*_2_ gene, making the fuzz density generally low and is often influenced by the environmental stimuli [37]. A recent study mapped the *N*_1_ to chromosome A12, which is thought to be a homologous gene of *MYB25-like* (*GhMML3_A12*) [14]. However, the *n*_2_ gene has still not been discovered, and the environmental stimulus affecting the function of the *n*_2_ gene remains unclear. In this study, we explored the effect of temperature on *G. barbadense* fuzz density and speculated that *n*_2_ might be temperature-regulated. Therefore, L7009 is likely to be an island cotton variety carrying the *n*_2_ gene, and the *n*_2_ gene will be identified from L7009 by QTL or QTL-seq mapping technology in the future. Cotton breeders interested in the genetics of fuzz initiation and the *N*_1_ and *n*_2_ loci in G. barbadense could be mapped by making crosses and populations with L7009 to confirm that it is allelic or new loci.

### 4.2. Transcriptome Sequencing Revealed the Expression Pattern Related to Fuzz Fiber Initiation

So far, many studies on fiber initiation have been carried out using intact cotton ovules [29,49,61], because it is difficult to separate the surface layer manually or initial fiber cells (including fuzz and lint) from the ovules. However, these studies using the whole ovule to reveal the mechanism of fiber initiation development ignore the interference of other tissues of the ovule. To tackle this problem, Haiyan et al. [62] used a stereomicroscope to cut the surface layer from the ovules and conducted transcriptome sequencing to mine genes related to fiber initiation. As we all know, the initial fuzz fibers are entangled with the lint fibers, and the separation of fuzz fibers or surface layer from the cotton ovules is also at risk of being interfered with by the lint fibers. Members of co-expressed gene modules usually have similar expression trends, and gene co-expression analysis is often used to obtain candidate gene sets related to specific developmental stages or important traits [63,64,65]. Based on the difference in the development process of lint fibers and fuzz fibers, an expression pattern closely related to the fuzz initiation process was revealed in our research. These co-expressed genes were considered to play an essential role in the fuzz fiber initiation induced by high temperatures. It is worth noting that whether the transcription and expression of these genes can directly trigger the fuzz initiation under high temperatures still needs further functional verification. With the rapid development of biotechnology, laser capture microscopy (LCM) coupled with global transcriptome profiling has been favored by scientists in the study of transcriptional regulation of cells in specific plant tissues [66,67,68], which may be one of the better candidate strategies to study the fuzz initiation induced by high temperatures in the future.

### 4.3. Major Transcription Factors Involved in Fiber Development

In cotton, many TF families have been studied, and a large number of TF genes participate in regulating the fiber development and plant responses to environmental factors. In the three major clusters, four TF families with the most genes are NAC, bHLH, MYB, and GRAS, whose members participate in the development of different types of fibers. NAC transcription factors have multiple functions in abiotic stress responses and plant development [69]. In the present study, sixteen genes belonging to the NAC TF family were recruited from the three important clusters, and six of these genes were mainly expressed in cluster 7. Of these, *GhNAC074* (*GB_A08G2284*) was the only one expressed at 4 dpa in the HT, *GhNAC047* (*GB_D11G2744*), and *GhNAC100* (*GB_A11G0737*) were predominantly expressed in H4, but with lower expression at other stages in both environments. The bHLH TF genes, *AtGL3* and *AtEGL3*, play redundant roles in *Arabidopsis* root hair regulation, and the double mutants have hairy roots [70]. As a bHLH protein, GhDEL65 can partially restore the trichome development of *Arabidopsis gl3 egl3* double mutants, and the overexpression in wild plants increase the trichome density of leaves and stems [51]. Our results identified two bHLH TF genes homologous to *AT2G40200*, *GB_A10G2822*, and *GB_D10G2781*, which were mainly up-regulated at 4 dpa in the high temperature environment (cluster 7). In addition to bHLH and NAC, members of MYB and C2H2 also play an important role in regulating cotton fiber development. As mentioned earlier, the MYB genes play an essential role in regulating the initiation and elongation of cotton fibers, such as *GaMYB2*, *GhMYB109*, *GhMYB25*, and *GhMYB25-like* [13,15]. In this study, two MYB genes homologous with MYB116 and *MYB305* were expressed in fuzz initiation stages, indicating that they play a role in regulating fuzz development. C2H2 genes have also been reported to play an important role in determining the epidermal cell fate [71]. As members of the C2H2 genes, *GIS*, *ZFP5*, and *ZFP6* promote trichome initiation by transcriptional activation of the downstream trichome initiation complex (GL1-GL3-TTG1) [72]. Our results showed that the only C2H2 gene *STZ* was highly expressed during fuzz initiation development in HT. Although few C2H2 genes have been reported to be involved in fiber development in cotton, there should be some functional homologous genes in cotton according to the regulatory similarity between cotton fibers and *Arabidopsis* trichomes.

### 4.4. Maintenance of ROS Homeostasis is Sufficient to Temperature-Regulated Fiber Initiation

Reactive oxygen species (ROS) mainly include superoxide radical, hydrogen peroxide, and hydroxyl radical, which can regulate plant cell proliferation and expansion, cell differentiation (such as root hair and lateral root), dedifferentiation, and tissue regeneration [73]. Ascorbate peroxidase (APX), a hydrogen peroxide scavenging enzyme, is involved in regulating ROS homeostasis [74]. The expression of *GhAPX1* at 5 dpa in the *fl* mutant ovule was much lower than that of the wild type, and the application of H_2_O_2_ induced the expression of *GhAPX1* to promote fiber elongation. Enzyme activity and H_2_O_2_ content assays showed that APX members, except *GhAPX1*, were mainly involved in the late fiber development (30 dpa) [75]. *GhPOX1* encodes a class III peroxidase that functions during fiber elongation by mediating ROS production [76]. Studies have shown that homeostasis of the H_2_O_2_ and redox levels is an indispensable mechanism that inhibits fiber initiation or elongation [77]. Increased H_2_O_2_ content was detected in 5 dpa ovules of cotton, and H_2_O_2_ could promote fuzz initiation in vivo [78]. In our study, we found that a portion of the fuzz fiber initiation related candidate genes were significantly enriched in the oxidative stress response pathway, in which two hydrogen peroxide scavenging enzyme genes, *GbPrx52* and *GbRCI3*, were found. In *Arabidopsis*, *AtPrx52* is involved in lignin synthesis as a hydrogen peroxide scavenging enzyme, while *AtRCI3* is primarily involved in tolerance regulation under various abiotic stresses [79,80]. In this study, the content of H_2_O_2_ and the activity of antioxidant enzymes at 4 dpa in the HT environment were lower than that in LT, indicating that the ROS levels in the HT environment were maintained at a suitable state for the fuzz initiation development, and the ROS homeostasis in LT environment might be disrupted to a certain extent, which led to the failure of the fuzz initiation. In summary, it is believed that the ROS homeostasis regulation plays a key role in the fuzz initiation period.

## 5. Conclusions

This study emphasized that thermal stimulus has a significant effect on lint and fuzz fiber development, and proved that temperature determines the fuzz initiation of a *G. barbadense* variety L7009. Low temperatures could inhibit the fuzz initiation and delay the lint fiber development, while high temperatures could relieve the negative effects of low temperature on these phenotypes. Through comparative transcriptome analysis, we identified a large number of DEGs (9667) involved in both fiber development and temperature response, obtained and three expression patterns closely related to the different development of the fibers—of which the genes in cluster 7 were differentially expressed between the two temperature environments during the fuzz fiber initiation. Functional enrichment analysis indicated that DEGs involved in the asparagine biosynthetic process, cell wall synthesis, and stress response were potentially related to the fuzz initiation induced by high temperature. Transcription factors, plant hormone signals, and ROS homeostasis maintenance may be possibly involved in fuzz initiation at high temperatures. These findings can help us understand the morphology and molecular mechanism of fuzz initiation regulated by high temperature in *G. barbadense*.

## Figures and Tables

**Table 1:** Summary of sequencing data for different fuzz fiber developmental stages in high and low temperatures.

Sample	Raw Reads (×10^7^)	Raw Bases (×10^9^)	Clean Reads (×10^7^)	Clean Bases (×10^9^)	GC (%)	Q30 (%)	Mapped Reads (%)
H1-1	4.26	6.38	4.18	6.27	45.94	93.71	92.62
H1-2	4.15	6.22	4.07	6.10	45.63	93.83	94.86
H4-1	4.23	6.34	4.14	6.22	46.19	93.46	94.49
H4-2	4.19	6.28	4.11	6.16	45.81	93.61	95.17
H7-1	4.11	6.17	4.03	6.05	46.13	93.32	94.54
H7-2	4.25	6.37	4.17	6.25	45.80	93.56	94.89
L1-1	4.10	6.16	4.02	6.03	45.25	93.50	91.86
L1-2	4.10	6.15	4.01	6.01	45.54	93.62	90.62
L4-1	4.14	6.21	4.05	6.08	46.98	93.30	92.65
L4-2	4.17	6.25	4.09	6.13	45.76	93.45	94.36
L7-1	4.17	6.25	4.08	6.13	45.92	93.33	95.05
L7-2	4.08	6.11	4.00	6.00	45.84	93.56	92.36

**Table 2:** Loop comparisons of the differentially expressed genes within and between the two environments at three stages during fuzz fiber development.

Comparison	Total	Up-Regulated	Down-Regulated
H1-vs.-H4	14,490	3393	11,097
H1-vs.-H7	12,716	3628	9088
H4-vs.-H7	2160	1230	930
L1-vs.-L4	5694	1473	4221
L1-vs.-L7	17,748	3352	14,396
L4-vs.-L7	4290	1667	2623
L1-vs.-H1	2480	951	1529
L4-vs.-H4	7355	3072	4283
L7-vs.-H7	4164	2876	1288

## Supplementary Materials

The following are available online at https://www.mdpi.com/2073-4425/11/9/1066/s1, Figure S1: Visual grading of fuzz density, Figure S2: Correlation between fiber quality and fuzz density, Figure S3: Cotton fiber quality developed in development temperature environments measured by AFIS and HVI, Figure S4: Venn diagram of DEGs from different comparisons, Figure S5: Clusters of expression patterns of DEGs involved in both temperature response and fiber development, Figure S6: Treemap of GO-terms enriched in cluster 5 and 6, Figure S7: The relative expression of sixteen randomly selected genes examined by qRT-PCR, Table S1: Fuzz fiber density and lint fiber quality of 121 *G. barbadense* varieties, Table S2: Matrix of significance values between the same stage under different treatments, Table S3: Alternative splicing prediction and novel genes annotation, Table S4: The enriched GO-terms of the three major clusters, Table S5: KEGG enrichment of genes in the three major clusters, Table S6: Differentially expressed transcription factor genes of the three major clusters, Table S7: DEGs involved in plant hormone signal transduction pathway in the three major clusters, Table S8: Primers for qRT-PCR, Table S9: Pearson correlation coefficients between the nine Mfuzz clusters.
