# Supplementary material for: Transcriptome Analysis Reveals a Gene Expression Pattern Associated with Fuzz Fiber Initiation Induced by High Temperature in Gossypium barbadense
Source: Genes (Basel). 2020 Sep 10;11(9):1066. doi: 10.3390/genes11091066 (PMC7565297; doi:10.3390/genes11091066)
Supplement: Supplementary file 1 [file genes-11-01066-s001.zip › Supplementary Files/Figure S2.pdf]

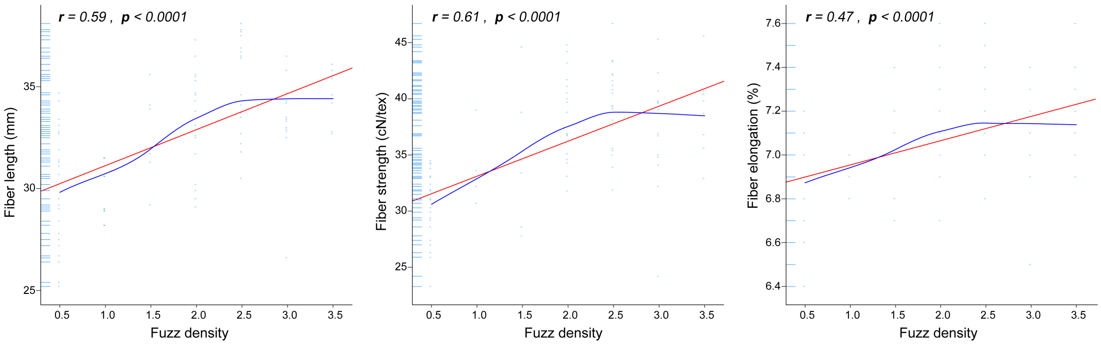

Figure S2. Correlation between fiber quality and fuzz density. The correlation coefficient is given by *r*-value, and the correlation significance is marked by *p*-value. The data came from 121 island cotton varieties planted in the winter of 2016 in Sanya, Hainan province. The fiber quality was measured by High Volume Instrument (HVI), and the fuzz density was graded by manual visual measurement.
