# Supplementary material for: Transcriptome Analysis Reveals a Gene Expression Pattern Associated with Fuzz Fiber Initiation Induced by High Temperature in Gossypium barbadense
Source: Genes (Basel). 2020 Sep 10;11(9):1066. doi: 10.3390/genes11091066 (PMC7565297; doi:10.3390/genes11091066)
Supplement: Supplementary file 1 [file genes-11-01066-s001.zip › Supplementary Files/Figure S3.pdf]

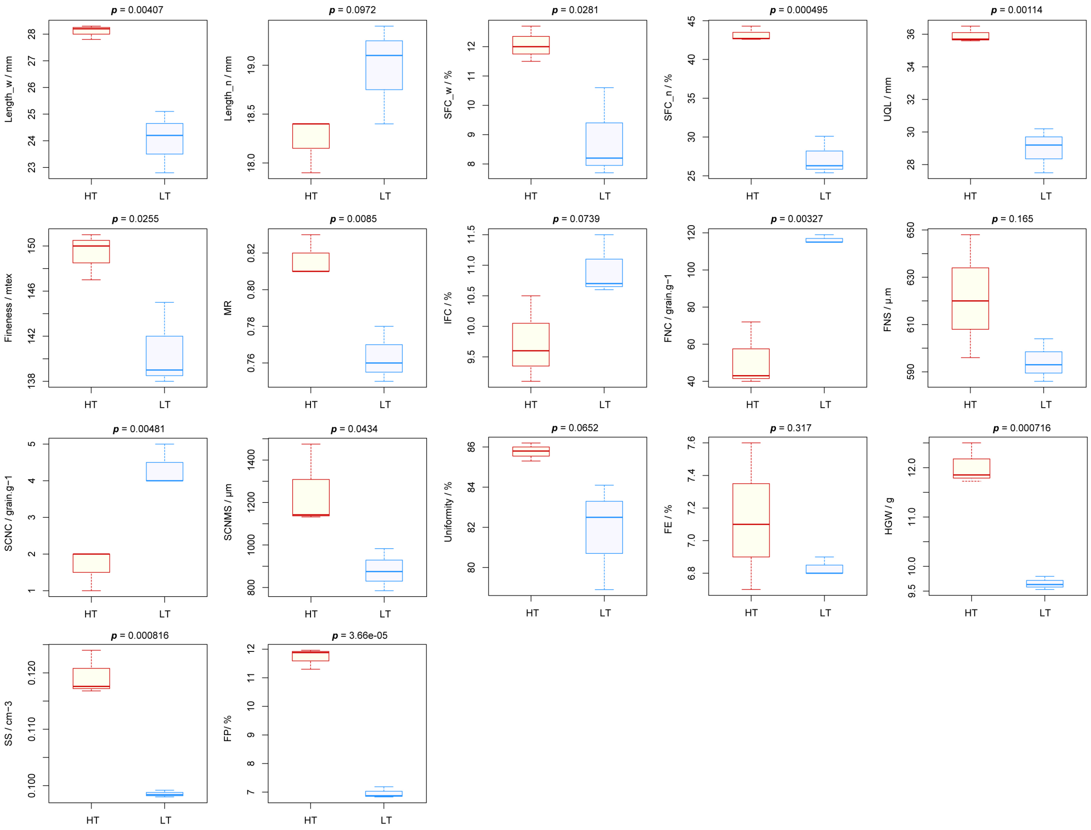

Figure S3. Cotton fiber quality developed in development temperature environments measured by AFIS and HVI. Twelve fiber quality traits measured by Advanced Fiber Information System (AFIS): length based on weight (Length_w), length based on number (Length_n), short fiber content based on weight (SFC_w), short fiber content based on number (SFC_n), upper quartile length (UQL), Fineness, mature ratio (MR), IFC (Immature fiber content), fiber neps content (FNC), FNS (fiber neps size), seed coat neps content (SCNC), and seed coat neps mean size (SCNMS). The fiber quality trait of Uniformity and fiber elongation (FE) measured by HVI. Three seed traits, such as 100-grain weight (HGW), seed size (SS), and fuzz percentage (FP), were determined by artificial methods. The significant difference was represented by *p*-value.
