# Supplementary material for: Transcriptome Analysis Reveals a Gene Expression Pattern Associated with Fuzz Fiber Initiation Induced by High Temperature in Gossypium barbadense
Source: Genes (Basel). 2020 Sep 10;11(9):1066. doi: 10.3390/genes11091066 (PMC7565297; doi:10.3390/genes11091066)
Supplement: Supplementary file 1 [file genes-11-01066-s001.zip › Supplementary Files/Figure S5.pdf]

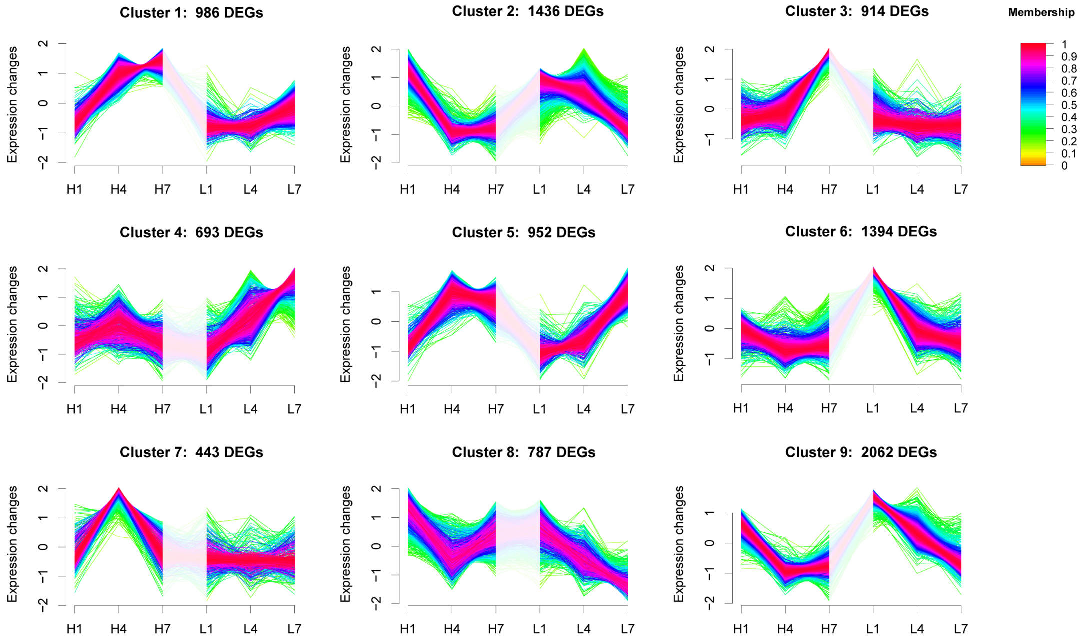

Figure S5. Clusters of expression patterns of DEGs involved in both temperature response and fiber development. The change of expression level was shown after Z-score normalization. The Mfuzz program of R v. 3.6.2 software was used for clustering analysis.
