# Supplementary material for: Transcriptome Analysis Reveals a Gene Expression Pattern Associated with Fuzz Fiber Initiation Induced by High Temperature in Gossypium barbadense
Source: Genes (Basel). 2020 Sep 10;11(9):1066. doi: 10.3390/genes11091066 (PMC7565297; doi:10.3390/genes11091066)
Supplement: Supplementary file 1 [file genes-11-01066-s001.zip › Supplementary Files/Figure S6.pdf]

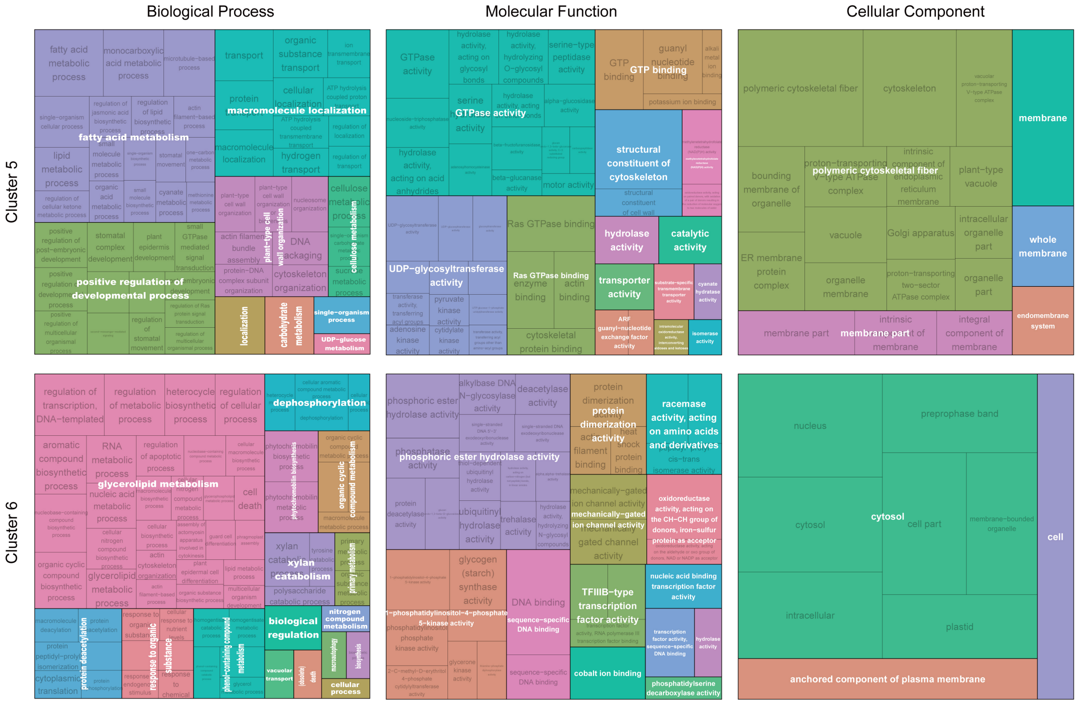

Figure S6. Treemap of GO-terms enriched in cluster 5 and 6. In each treemap, rectangles represent significant GO-terms. GO-terms sharing the same colour belong to the same GO superclusters whose names are labelled in white colour. The sizes of rectangles reflect the relative corrected *p*-value (the larger the rectangle the more significant the GO-term).
