# Supplementary material for: Transcriptome Analysis Reveals a Gene Expression Pattern Associated with Fuzz Fiber Initiation Induced by High Temperature in Gossypium barbadense
Source: Genes (Basel). 2020 Sep 10;11(9):1066. doi: 10.3390/genes11091066 (PMC7565297; doi:10.3390/genes11091066)
Supplement: Supplementary file 1 [file genes-11-01066-s001.zip › Supplementary Files/Figure S7.pdf]

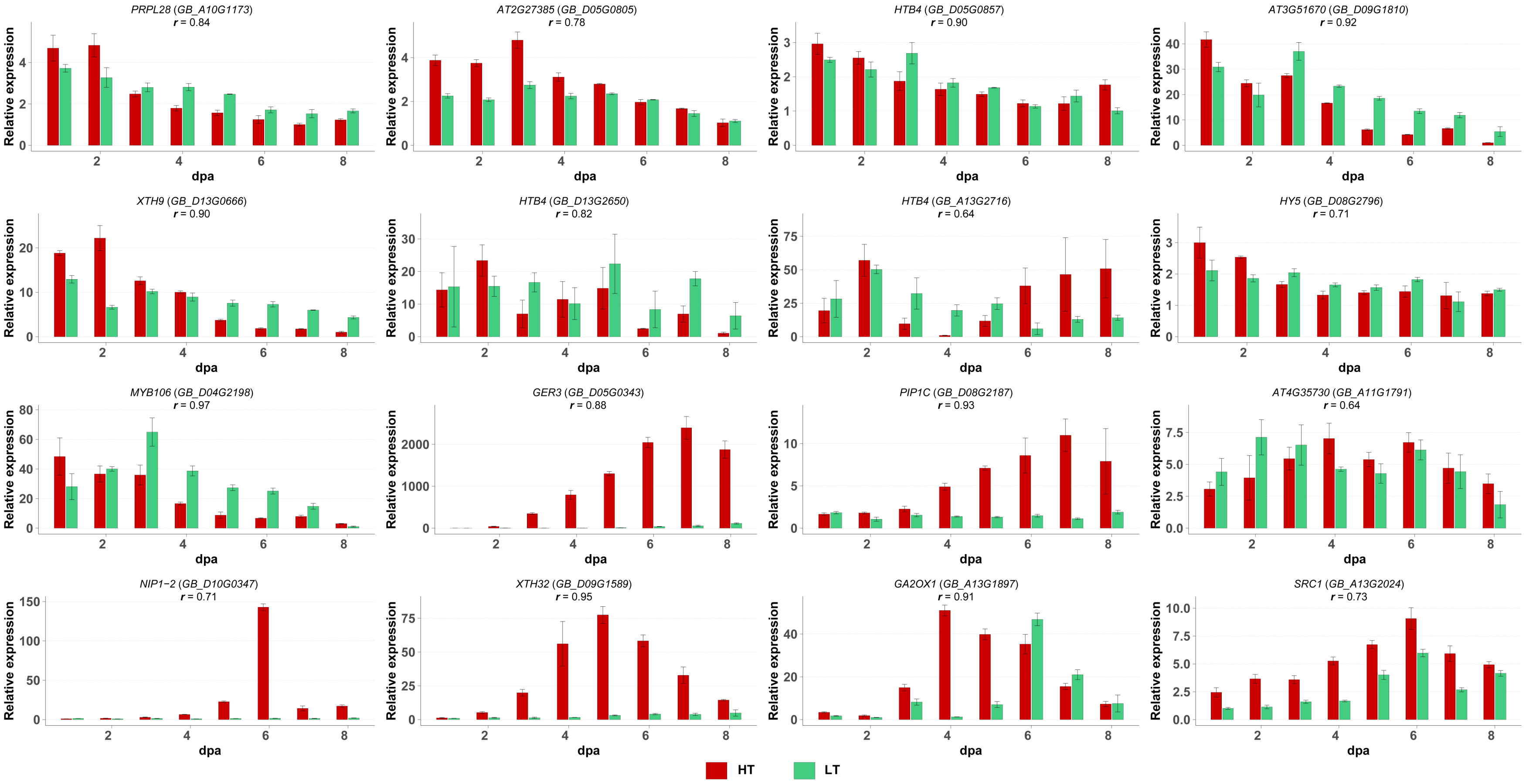

Figure S7. The relative expression of sixteen randomly selected genes examined by qRT-PCR. Correlation coefficients between RNA-seq data and qRT-PCR expression data were correspondingly represented by *r*-values. All values were shown with mean ± SE.
