# Supplementary figures and images for: Transcriptome Analysis Reveals a Gene Expression Pattern Associated with Fuzz Fiber Initiation Induced by High Temperature in Gossypium barbadense
Source: Genes (Basel). 2020 Sep 10;11(9):1066. doi: 10.3390/genes11091066 (PMC7565297; doi:10.3390/genes11091066)

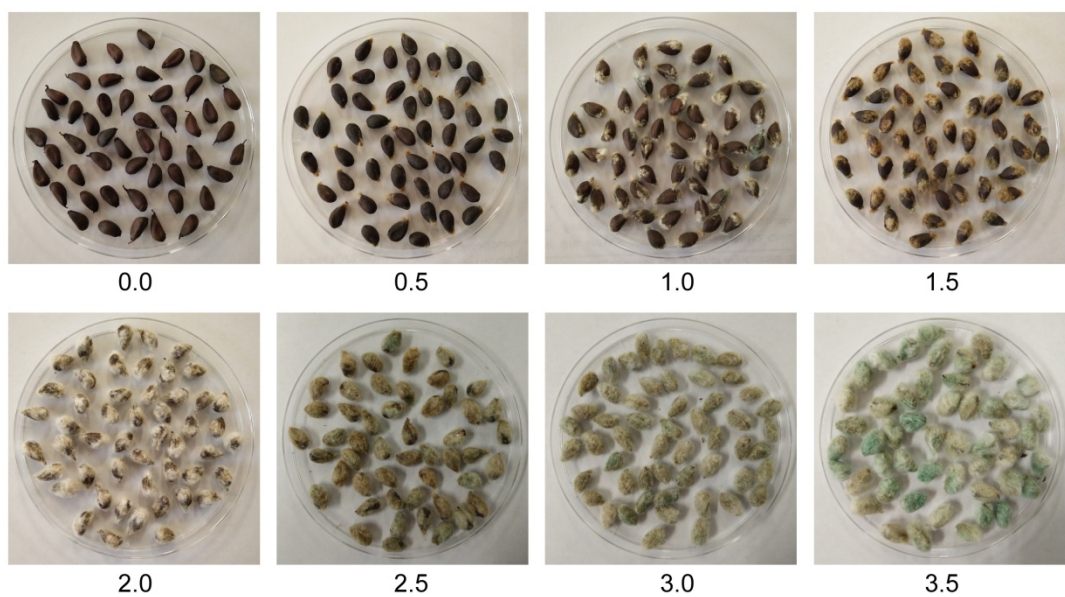

**Figure S1.** Visual grading of fuzz density.

Supplement: Supplementary file 1 [file genes-11-01066-s001.zip › Supplementary Files/Figure S1.pdf]

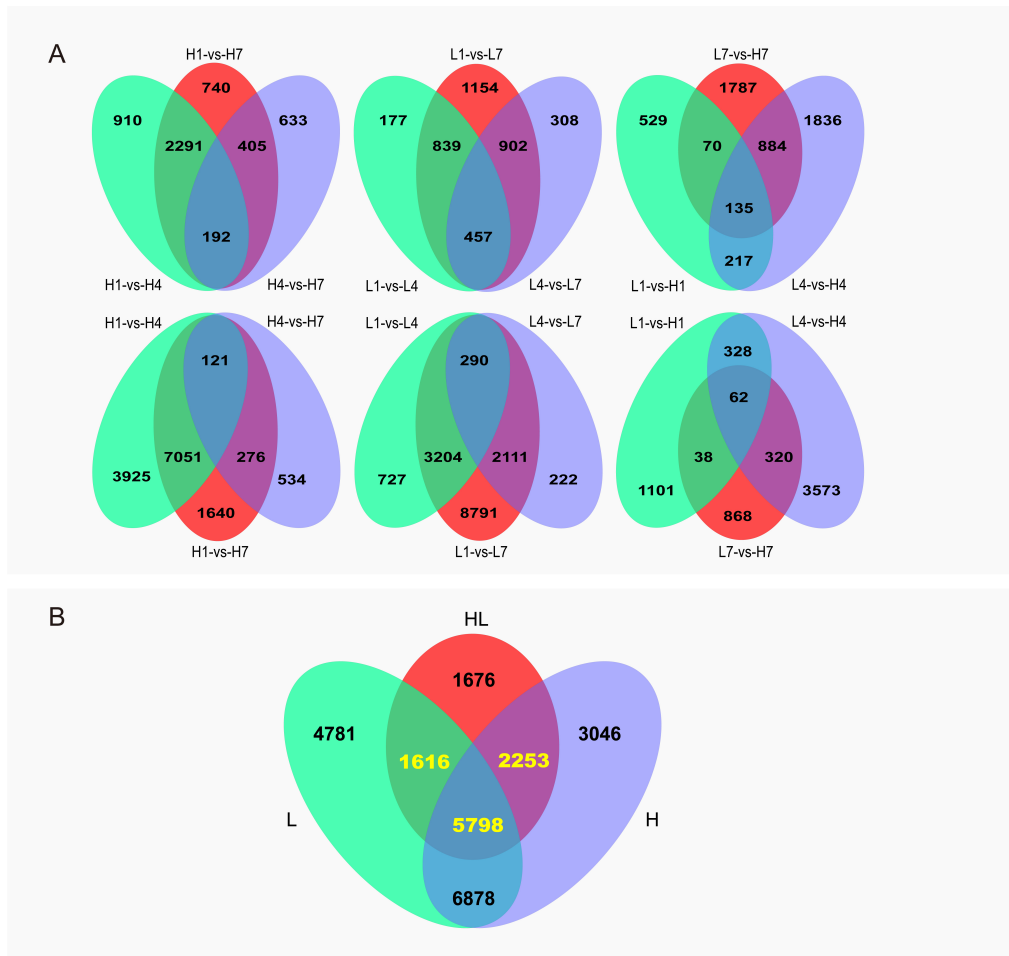

Supplement: Supplementary file 1 [file genes-11-01066-s001.zip › Supplementary Files/Figure S4.pdf]
